# Think Melkersson-Rosenthal Syndrome: A Fissured Tongue With Facial Paralysis

**DOI:** 10.7759/cureus.9480

**Published:** 2020-07-30

**Authors:** AbdulRahman Ibrahim, Moayed Ibrahim, Mohammad Al Adawi, Liyana Oweis, Yacoub Bahou

**Affiliations:** 1 Neurology, University of Jordan, Amman, JOR; 2 Hematology and Medical Oncology, Tulane University School of Medicine, New Orleans, USA; 3 Internal Medicine, University of Jordan, Amman, JOR

**Keywords:** melkersson-rosenthal syndrome, recurrent, facial nerve palsy

## Abstract

Melkersson-Rosenthal syndrome (MRS) was first described and named after E. Melkersson in 1928 and C. Rosenthal in 1931. MRS is a rare cause of recurrent facial nerve palsy and can manifest as facial paralysis, orofacial edema, and/or tongue fissuring. Presenting with the complete triad, it was scarcely reported in literature. However, the patient reported here had the complete triad. MRS should be considered when facial paralysis is recurrent or when it presents with orofacial edema, and/or tongue fissuring.

## Introduction

Bell's palsy has many differential diagnoses, one of which is Melkersson-Rosenthal syndrome (MRS), a rare cause of recurrent facial nerve palsy with a female predominance. It presents with facial paralysis, orofacial edema, and tongue fissuring [1]. The etiology of this syndrome is complex and multifactorial, resulting from the interplay of genetic, infectious, and immunologic factors [2]. Incomplete presentations outnumber the classic triad. The case presented here manifested with the complete triad, which is scarcely reported in literature.

## Case presentation

**Figure 1:**
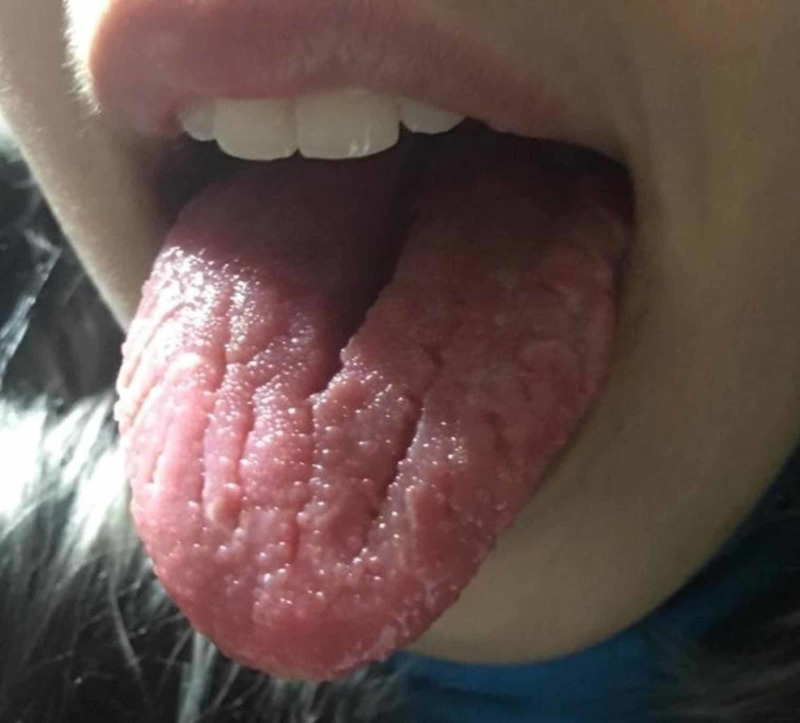
Tongue fissuring was prominent.

A 21-year-old female patient presented to the neurology clinic with recurrent episodes of left-sided mouth deviation, unilateral ptosis, ipsilateral blurred vision, dysphagia, jaw claudication, and upper lip swelling. This happened four times over the year prior to presentation, with alternation in the sides affected. She reported having her first episode at the age of eight years. Physical examination revealed loss of facial wrinkling on the right side, drooping of the right corner of the mouth, facial edema mainly in the upper lip, and tongue fissuring (Figure 1). The patient could not close her right eye on resistance, and could not reveal her teeth properly, demonstrating a lower motor neuron lesion in the right facial nerve. Her hearing was intact in the previous and current episodes. Laboratory tests were unremarkable for blood count, electrolytes, liver, and kidney tests. Serum IgM and IgG for Borrelia burgdorferi were negative, ruling out Lyme disease. Testing for herpes simplex virus, herpes zoster virus, and brucellosis was not done due to a low suspicion given lack of associated clinical signs. 

**Figure 2:**
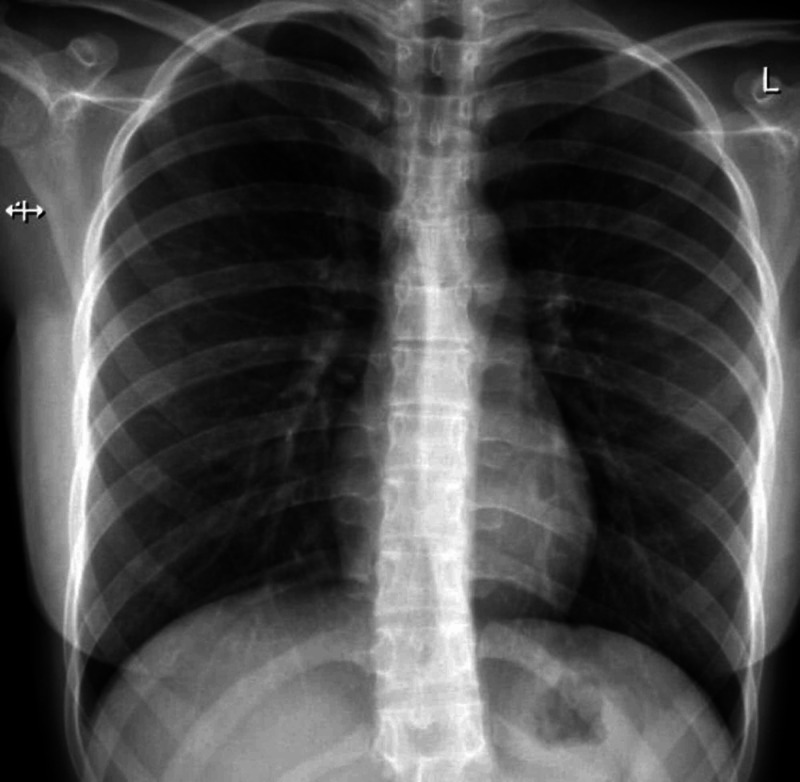
Chest Xray was unremarkable.

Chest X-ray (Figure 2) did not show any lung lesions. Sarcoidosis was not suspected, especially without a rash, cough, lymphadenopathy, fever, or weight loss. Therefore, angiotensin-converting enzyme (ACE) level was not checked. 

**Figure 3:**
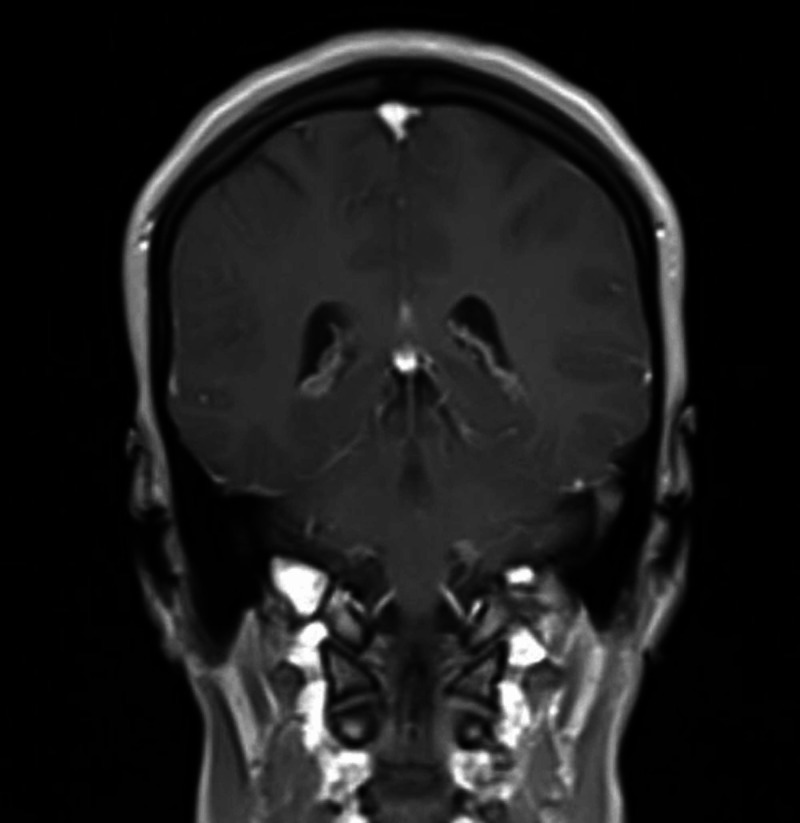
Brain MRI with and without contrast was unremarkable.

Brain MRI with and without contrast (Figure 3) was unremarkable. Lumbar puncture was contemplated but eventually not done due to lack of meningeal signs, unilateral presentation, and recurrence of these episodes. The diagnosis of MRS was made after excluding the relevant differential diagnoses of recurrent Bell's palsy. There is not a proven treatment for MRS. Our patient did not recall the treatment modalities of her prior episodes. She was started on azathioprine and prednisone. The patient responded completely to within a few weeks and continues to be symptom free. 

## Discussion

MRS is a rare disorder that uncommonly presents with the classical triad of orofacial edema, facial never palsy, and tongue fissuring [1,2]. The reported patient had the triad on the most recent episode, which clued us to MRS. Before confirming the diagnosis of MRS, other potential causes of recurrent facial nerve paralysis have to be ruled out, since it was the dominant feature in our case [3,4]. Brain MRI was sufficient to rule out neurosarcoidosis. Lyme disease was ruled out by serology. The most common finding of MRS is acute, diffuse, painless, and nonpitting orofacial edema that is most often present on the lips, with upper lip predominance [2]. Edema is usually recurrent and disappears within a few hours to a few weeks [5]. Our patient had four episodes of recurrent orofacial edema mostly presented on the upper lip during the past year, concurrent with episodes of facial nerve palsy. Edema is caused by impaired lymphatic drainage as demonstrated by lymphoscintigraphy studies [6]. Interestingly, lymphoscintigraphy after treatment of MRS shows restoration of normal lymphatic drainage of the affected areas.

The second characteristic finding of MRS is recurrent facial paralysis, which can present unilaterally or bilaterally. It has been proposed that the paralysis is caused by either the compressive effect of the edema on the facial nerve while it is crossing the facial canal or due to the granulomatous infiltration of the nerve [7]. Our patient had four episodes of alternating facial palsies in the last year, with a history of a previous episode at age of eight years. All of these episodes were associated with orofacial edema. The third characteristic finding of MRS is tongue fissuring, which was also prominent in our patient. Management of MRS chiefly targets relieving the orofacial edema by intralesional or systemic corticosteroids administration, which usually achieves a complete resolution of the edema [8]. Surgical excision and reconstruction are sometimes employed when chronic lip edema persists [9]. As for the facial paralysis, it has been reported that most patients have spontaneous recovery in three weeks [10]. Our patient’s orofacial edema, tongue fissuring, and facial paralysis completely resolved after administration of systemic corticosteroids and azathioprine.

## Conclusions

MRS is a rare syndrome and presenting with its three characteristic features simultaneously is uncommon. Although this increases the suspicion of MRS, other possible causes of recurrent facial paralysis should be ruled out first (e.g. neurosarcoidosis, Lyme disease, among others). Severity and time interval between the relapses vary, but orofacial edema seems to increase and persist longer with each episode. Management for all forms consists of nonsteroidal anti-inflammatory drugs (NSAIDs) and immunosuppressants.
